# “Caught in the crossfire” – women veterans’ testimonies regarding excessively violent acts committed in combat zones

**DOI:** 10.3389/fpsyg.2024.1286813

**Published:** 2024-04-10

**Authors:** Nehama HaCohen, Dana Amir

**Affiliations:** ^1^The Culturally-Sensitive Clinical Psychology Program, Achva Academic College, Shikmim, Israel; ^2^The Psychology Department, Bar-Ilan University, Ramat-Gan, Israel; ^3^The Trauma & Identity in a Multicultural Lens Lab, Achva Academic College, Shikmim, Israel; ^4^The Briah Foundation for Women’s Health, Tel Aviv, Israel; ^5^The Interdisciplinary Doctoral Program in Psychoanalysis, Department of Counseling and Human Development, University of Haifa, Haifa, Israel

**Keywords:** violence, women veterans, testimonies, military psychology, identification with the aggressor, internalized oppression

## Abstract

As women in the Israeli Defense Forces (IDF) are increasingly placed in supportive and combat roles in active war zones, they routinely encounter and participate in violent acts. This study focusses on the centrality of gendered inequality and oppression as a factor that shapes not only women’s experience in the military but also their responses in cases of excessive violence. The goal of this study was to explore the ways women veterans of combat or combat-support units conceptualize their stance regarding violent acts which they either committed or witnessed in war zones. Using a qualitative approach, we analyzed the retrospective testimonies of 58 Israeli women veterans from the archives of an NGO that documents veteran combatants exposure to excessive violence. Most women explained their violent acts as inherent to the military system and culture, which in our analysis was categorized as examples of either internalized gender oppression or as identification with the aggressor. A smaller number of women described their attempts to protest, as they took a moral stance rooted in a feminine perspective. The three explanations revealed through the analysis of the testimonies reflect the inner tension experienced by many women in the military, as they navigate between two extreme positions, either as victims of male dominance, or as aggressors that are part of a powerful military system. In this study, gendered inequality provides a framework for analyzing the data. Thus, this study contributes to the theoretical knowledge and methodological approaches concerning violent situations in combat areas, focusing on the various ways in which women veterans subjectively and retroactively conceptualize their participation in and responses to violent acts.

## 1 Introduction

Women’s struggle for equality has led to the expansion of roles available to women in the armed services, thus increasing their exposure to – and participation in – excessive use of violence, in a variety of complex and morally-troubling experiences (Vogt et al., 2011; Street et al., 2013). The literature about women in the military has typically focused on stressors unrelated to the battlefield, such as exposure to sexual trauma (Maguen et al., 2012; Brownstone et al., 2018; Dardis et al., 2018) or PTSD (Street et al., 2013), while research on men in the military has focused on combatant experiences, such as exposure to combat-related trauma (e.g., Freedy et al., 2010; Vogt et al., 2011) and other consequences, such as moral injury (Richardson et al., 2020). However, little research has been undertaken on women’s involvement in – or coping with – acts of excessive violence, in the context of combat or combat-support roles in war zones (Conway 2013; Kelley et al., 2019). The present study aims to help fill this gap, by analyzing women veterans’ testimonies about their encounters with acts and situations characterized by excessive violence, during their combat service, and about their varied, subjective conceptualizations of these experiences. In doing so, we hope to shed light on women’s stance regarding their participation in or abstention from excessively aggressive and violent acts in the military. We choose to zoom in on the internal dynamics and internal mental structures of women soldiers operating in war zones, as these are reflected in their narratives. By analyzing testimonies of 58 women soldiers in Israel, we were able to identify three main categories: identification with the aggressor, internalization of oppression, and overt resistance. We argue that these categories reflect the complex interplay of gender oppression and the institutionalized use of violence in the military, highlighting how women soldiers navigate their roles as both victims and aggressors. Our theoretical argument centers on the intersectionality of gender and military culture, highlighting how these unique experiences of female combatants impact their responses as well as their psychological well-being. In doing so, we propose a new direction in clinical psychology and in the psychology of trauma, which focuses on the unique needs of women soldiers and veterans. In what follows, we discuss the literature on the psychological effects of combat experience on soldiers, gender integration in the military, and the dynamics of oppression and resistance in the military with a specific emphasis on women’s experiences in these areas.

### 1.1 Female military personnel and masculinity

The military has long been a bastion of masculine ideology, where the values of strength, dominance, and emotional restraint are not only encouraged but deeply embedded within the culture and practices of the institution. Abraham et al. (2017) and Ramon et al. (2020) point out that masculinity ideologies in the military, which are distinct from biological sex, emphasize self-reliance and emotional control. This framework allows for the construction of a specific form of masculinity that all members, including women, are encouraged to emulate, creating an environment where traditionally masculine traits are rewarded. This emphasis on masculinity is further reinforced by socialization processes within the military that promote a particular brand of masculinity, focusing on strength, dominance, and emotional restraint as core values (Arkin and Dobrofsky, 1978; Braswell and Kushner, 2012; Basham, 2016).

The internalization of these masculine norms through social learning processes, such as becoming a combat soldier, plays a crucial role in maintaining gender-related power relations within society (Addis et al., 2010). Women in the military, particularly those in combat roles, face the expectation to conform to these traditional masculine norms, leading to a range of mental health-related outcomes including higher levels of PTSD and complex PTSD symptoms as a result of conforming to these norms through maladaptive coping strategies (Neilson et al., 2020; Zerach, 2023). This underscores the psychological toll that the militarized masculine culture can exert on women, highlighting the intricate relationship between gender conformity and mental health within this context.

Despite these challenges, the experience of female military personnel is not uniformly disempowering. Crowley and Sandhoff (2017) and Daphna-Tekoah and Harel (2023) explored the complex dynamics of gender roles in the military, noting how female combatants navigate the tension between empowerment and disempowerment. This negotiation reveals a multifaceted experience where, alongside exposure to trauma, women also feel valued and empowered by their service. This juxtaposition underscores the complexity of gender roles within the military, where empowerment can coexist with challenges stemming from a masculine-dominated environment.

The cultural and workplace dynamics within the military further complicate the experiences of female soldiers. Koeszegi et al. (2014) and Harel-Shalev et al. (2017) contrast the cultures of combat and support units, noting that the traditional masculine norms and high power orientation of combat units make women more vulnerable to workplace aggression and bullying, while support units exhibit a more positive attitude toward female soldiers. The military constitutes also a complex occupational field for women as one in which embodied masculinity is legitimized and rewarded, and women’s bodies are often perceived as problems to the extent that they deviate from this masculine standard and constructed as second-class workers (Steidl and Brookshire, 2019). This differentiation highlights the variance in how gender norms and power dynamics manifest across different military contexts, affecting the experiences of women in the army.

Lastly, the resistance to hegemonic masculinity within the military indicates a potential shift toward a more inclusive understanding of masculine identity. Robinson Kurpius and Lucart (2000), Perez and Sasson-Levy (2015), and Pendlebury (2020) highlight how individuals challenge rigid gender norms, advocating for a broader interpretation of masculinity. This resistance, alongside the role of language in reflecting and reinforcing gender biases (Smith et al., 2019; van Douwen et al., 2022), suggests an evolving military culture that is slowly accommodating more inclusive identities and norms.

The military, as an institution deeply rooted in masculine culture, presents a complex landscape for both men and women. The challenges faced by women in the army stem from a culture shaped by male norms, where the negotiation of gender roles and identities takes place within a framework that traditionally emphasizes and rewards masculinity. This dynamic not only impacts the experiences and mental health of female personnel but also reflects broader societal norms and the potential for their transformation within one of society’s most traditionally masculine institutions.

Building on this foundation, the present study seeks to reveal the less-discussed experiences of war-zone combatants, focusing on their attitudes about engaging in or witnessing violent events, and how these experiences are perceived and interpreted by female soldiers. Studies have extensively examined the implications and psychological consequences of engaging in or witnessing violent events within war zones, particularly focusing on how these experiences affect soldiers’ civil life (Mac Manus et al., 2015). A significant portion of the literature has centered on veterans’ and active soldiers’ PTSD, aggressive behaviors in the context of intimate partner violence, domestic violence (Taft et al., 2011; Hodges et al., 2022), and violence toward others in their environment (Straud et al., 2022). These studies elucidate the complex psychological landscape that soldiers navigate post-deployment, highlighting the variance in the nature of violence—ranging from aggression toward intimate partners to acts of violence against enemy combatants or civilians during battle (Maguen et al., 2012).

This exploration of violence in military contexts underscores the necessity of differentiating between types of aggression encountered by soldiers. Meyer-Parlapanis and Elbert (2015) argue that in each instance of violence, combatants must navigate the dual roles of perpetrators and survivors. Elbert et al. (2010) introduced a nuanced understanding of aggressive behavior during combat as a blend of reactive aggression—impulsive and provoked by perceived threats—and appetitive aggression, which is motivated by intrinsic reward and can be perceived as exciting. This distinction is crucial for understanding the adaptive strategies developed by soldiers in response to extreme stress and threats in violent environments (Crombach and Elbert, 2014; Hinsberger et al., 2016).

Given this multifaceted experience of combat and the evolving role of women in the military, it becomes imperative to closely examine the unique experiences and perspectives of female combatants. The conventional literature often overlooks these distinct experiences, highlighting a gap in our understanding of how gender roles and identities are negotiated and manifested within the context of military service and combat. By focusing on female combat soldiers, this study aims to enrich our understanding of the broader implications of military culture on individual experiences, particularly in the context of engaging in or witnessing violence.

### 1.2 Women in today’s military

Most literature on women in war zones has referred to them as victims rather than active agents (e.g., González-Castro et al., 2021). When it comes to considering women as combat soldiers, the literature still lags behind. Street et al. (2013) studied gender-specific war-zone experiences in a broad population of service members, representing all military branches. Although their results suggested that male and female service members’ military experiences tended to converge, they also found important gender differences in deployment stressors. These included women’s increased risk of sexual harassment, general harassment, and poorer unit support, as well as an increased risk of depression. Recent studies, which have compared men and women combat veterans, found that men and women veterans reported comparable levels of mental health symptoms (Kelley et al., 2019). Hence, the effects of combat service on men and women differ not in the degree of impact but in the areas of psychological struggle.

These findings suggest that the unique challenges faced by female combatants are not yet fully understood. The direction that this research field is mostly focused on is the integration of women into a masculine military environment. This direction is supported by the findings of Kelber et al. (2021) that higher rates of new PTSD diagnoses among women were not dependent on combat exposure, suggesting that other types of trauma (e.g., military sexual trauma) might have been responsible for these higher rates. It is becoming increasingly clear that the army’s hierarchical structure and the fact that women are a minority among soldiers, especially in leadership positions (Castro et al., 2015), often lead to their belittlement, objectification, and sexual harassment.

Sasson-Levy’s (2003) was the first to argue that Israeli women soldiers in masculine roles shape their gender identities according to the hegemonic masculinity of the combat soldier. She showed how this adaptation occurs through three interrelated practices: mimicry of combat soldiers’ physical and discursive practices; distancing from traditional femininity; and trivialization of sexual harassment. It was Harel-Shalev and Daphna-Tekoah (2016) who sought to understand the stories of women combat soldiers. Their findings shed light on the unique challenges faced by female combatants, as they stressed that women combatants often face a “double battle.” Not unlike male combatants, there is the challenge to adequately perform their military duties (which may include exposure to combat trauma) yet beyond this, female combatants are also struggling to integrate into a masculine military environment. Recent studies supported this conceptualization of the double challenge, as female service members reported that males construe female bodies as a threat to military effectiveness (i.e., as weak, unclean, and distracting; Van Gilder (2019)). In other words, women’s professionalism in the military, as related to their physical and mental abilities, was questioned. In parallel, there is a higher risk of female soldiers experiencing sexual violence (Stander and Thomsen, 2016), because their bodies are objectified while working and operating in a male-dominated environment.

Given that women in combat roles face this dual challenge, their silenced injuries could well be compounded by additional factors. Researchers claimed that women are apt to encounter additional stressors and to experience the same stressors in different ways than men do (Street et al., 2009). This study is designed to further uncover the experience of women veterans who committed and witnessed acts of excessive violence during their military service, and to analyze their reactions to these instances, in light of their unique positionality in the military.

### 1.3 Women and violence

Meyer-Parlapanis et al. (2016), in their innovative study, compared appetitive aggression (the perception of perpetrated violence as fascinating and exciting) among male and female war combatants. They paid particular attention to the perception of perpetrated acts, distinguishing between acts committed with neutral or negative emotions and those committed with pleasure. They showed that females can be carried away by the violence in a manner similar to males. In a context in which it is considered situationally “appropriate” to directly perpetrate violence against others, and when males and females have perpetrated similar types of violent acts, both males and females are capable of experiencing aggression as fascinating or pleasurable. However, in contrast to males, the initial barriers to the onset of violence may be higher among women. Thus, despite women’s higher threshold for appetitive aggression, the context of military combat, coupled with the need to adapt to and survive in the male-dominant environment, effectively diminishes this gap (Augsburger et al., 2017).

According to the existing literature, it appears that women in combat roles express behaviors and experience mental consequences similar to those experienced by men in the same roles. However, recent studies have also shown that operating in a male dominant environment poses a separate challenge. Therefore, it stands to reason that the ways in which women interpret and assimilate these behavioral and mental consequences would differ from the stance taken by their male counterparts in relation to the same events and their impact.

### 1.4 The current study

Dodds and Kiernan (2019) argue that current literature on women veterans is dominated by the U.S. experience and does not fully explore the global complexity of women veterans, or the difficulties military service causes them. Israel is the only Western country that has compulsory conscription for both men and women. Military service in Israel lasts three years for men and two years for women. Women comprise 33% of the Israeli Defense Forces (IDF; Karazi-Presler et al., 2018), and about 17% of its combatants (Foreign Affairs and Defense Committee, 2023). Nevertheless, women comprise only 4 % of the colonels in the IDF (Daphna-Tekoah et al., 2021).

The Israeli-Palestinian conflict differs in many essential ways from other cases of collective violence. During the extremely violent period referred to as the “Second Intifada,” (2000–2005, the years referred to in the participants’ testimonials), more than 700 Israeli civilians and more than 300 members of the security forces were killed, as well as more than 4,000 Palestinians. Israeli security forces were at the eye of the storm, struggling to differentiate insurgency from innocent civil routine. This involved active policing in populated areas, including manning checkpoints and arresting individuals suspected of engaging in terrorist activity. Military orders were many times ambiguous and left the soldiers on the ground to confront highly complex and morally fraught situations. Given the perpetual threat of violence, soldiers had to operate under conditions of constant stress and fear over an extended period of time, during which they also experienced the loss of friends or relatives in combat or terror attacks. The psychological impact of contending with such conditions is inevitable, and may include numbing, demoralization, and disintegration of decision-making abilities (Zerach and Levi-Belz, 2018).

Military service in a war zone can lead to significant traumatization, including “moral trauma” (Maoz, 2001). Recent studies have indicated that violent acts are common among young Israeli veterans, with one third of the study population reportedly witnessing at least one event perpetrated by others, and more than one fifth reporting at least one self-perpetrated event (Zerach and Levi-Belz, 2018). As regards the impact of violent conditions and events on women in the Israeli military, the experiences and their psychological consequences are still unknown. Therefore, the testimonies of Israeli female veterans provide a unique opportunity for closing this knowledge gap. Specifically, this study examines how women describe acts of excessive violence, which they either committed or witnessed during their combat service or in combat support roles, and analyzes their responses from a psychological perspective.

Recently, Richardson et al. (2020) emphasized the need to study the voices of service members and/or veterans as they describe experiences that might have been silenced elsewhere. Also Daphna-Tekoah et al. (2021) recommended genuinely listening to female veterans’ experiences, to understand their unique struggles. Notably, researchers found that conventional research methods may be inadequate for capturing these experiences, due to the complex circumstances that characterize women’s combat-zone life. Our study attempts to compensate for this silence, by retrieving and analyzing female military veterans’ testimonies about their experiences of excessively violent acts witnessed or committed while serving in the IDF. In their testimonies, the participants focused on their actions in combat or combat-support roles.

While the onset of post-traumatic symptoms among those who have served in combat has been extensively studied and occupies a prominent position in trauma research (Xue et al., 2015), the internal psychological experiences of veterans have received comparatively less attention. Studies that have delved into the psychological and emotional experiences of discharged combat soldiers suggest a pervasive sense of loneliness (for a review, see Wilson et al., 2018). Stein and Tuval-Mashiach (2015) characterized this as “experiential isolation,” manifesting as a perceived unbridgeable gap between their vivid experiences and those of others around them. This feeling is often summarized by the expression “A stranger cannot fathom it,” suggesting that only those who shared the same experiences can truly understand. In this study, we argue that this might also contribute to the difficulty of researching these experiences. When it comes to sharing one’s experience of violent acts, the bond of silence often intensifies. This study progresses by examining the experiences of women who have served in combat zones, as narrated to interviewers who themselves are female combat veterans. We assumed that in this context, these women would feel more understood and thus be able to express their experiences in a more sincere and nuanced manner, particularly concerning issues related to the use of excessive violence.

This study is premised on the dual challenges women combatants face in performing military duties and their struggle to integrate into the masculine military environment (Harel-Shalev and Daphna-Tekoah, 2016), coupled with the intricate balance all combatants must maintain between perpetration and survival during violent encounters (Meyer-Parlapanis and Elbert, 2015).

The aim of the current study was to explore how women veterans make sense of and attribute meaning to their reactions to (i.e., becoming involved in, refraining from, or expressing opposition to) acts of excessive violence encountered during military service in war-zones. To this end, we analyzed women veterans’ testimonies, which we treated as the arena in which the meaning-making process occurs. Specifically, these testimonies reflected participants’ attempt to resolve the inner conflict aroused by morally fraught situations (Litz et al., 2009).

In this context, we asked: How do women veterans conceptualize their stance regarding violent acts that they actively or passively participated in, and how do they make sense of these situations?

## 2 Materials and methods

This study analyzes testimonies of female military veterans about their service in the IDF in a war zone, either in Gaza or the West Bank. The focus of the testimonies was on the women’s actions as officers, commanders, or soldiers, in combat or combat-support roles. The data for the study were taken from the archives of “Breaking the Silence” (BtS), an Israeli NGO that collects testimonies in which Israeli veterans (men and women) talk about their compulsory army service in the West Bank and Gaza Strip. Here, we reviewed testimonies of female officers, commanders, and soldiers who approached the BtS organization of their own initiative, as a moral-political act, and described how they dealt with complex situations on the ground. All the testimonies in the current study, which were compiled by BtS activists who too are women veterans, described military experiences accumulated during the years 2000–2010.

This unique database provides an authentic and rare glimpse into the way military reality is conducted and within it, the choices the soldiers and military personnel make and the way they explain them. In contrast to interviews, which are conducted for the purpose of research, the testimonies in the BtS database are sometimes intended to provide a confessional outlet, where people can document immoral acts they committed or witnessed during their military service and now are willing to voice them publicly. Their goal is both to obtain personal moral relief and to expose facts that are likely to promote public discussion, which eventually could lead to a change of policy.

Approximately at age 18, the interviewees had been enlisted into the IDF for 2 years of compulsory military service; the interviews were conducted approximately 5 years after their discharge. All those who volunteered to give testimony were fully informed of the organization’s aims. The interviews were audio-recorded and transcribed, and printed in their original form, excluding details that might disclose the testifier’s identity. For this study, we analyzed one booklet titled, *Women Breaking the Silence* (WBtS), which included 96 testimonies. The study did not require IRB approval in Israel, as the materials used were publicly published in the media, and furthermore, upon providing the testimony, the testifiers gave their explicit permission for the material to be used for research purposes.

Conceptualizing the experience of committing violent acts and exploring the related meaning-making processes presents a methodological challenge, as it is inherently difficult to assess such a complex phenomenon. However, previous studies that used retrospective testimonies to study perpetrators’ narratives have proven to be valuable in revealing authentic evidence that expanded our understanding of these inaccessible areas of research (Meyer-Parlapanis et al., 2016; Amir and HaCohen, 2020, 2021).

### 2.1 Data collection

In the interviews, participants were asked a series of questions regarding their military service in a war zone. We were interested in women veterans’ reflections on their own position, as witnesses of or participants in violent acts in the military. Therefore, we limited the scope of this study to testimonies in which the speakers referred to a moral dilemma, related either to their own immoral actions, or to ones they had witnessed firsthand. From the original 96 testimonies, we excluded 23 testimonies which referred to events that were not directly related to the testifier, resulting in 73 analyzed testimonies. A total of 122 pages of interview transcripts were fully analyzed.

We received the testimonies numbered and anonymous, identified only by the interviewee’s role in the military and the geographic location where she served.

### 2.2 Analytic procedures

Analyzing narratives is particularly useful for studying violent acts and violent situations, because the stories that are told indicate the significance that the speaker attributes to his/her life and the events experienced. The stories and the act of storytelling reveal not only the speaker’s self-perception but also the process of developing the narrative and giving meaning to the content (McAdams, 1993).

Grounded theory is used to develop a theory based on the experiences participants share about their lives (Fassinger, 2005) and can be understood within a framework of hermeneutic analysis (Rennie, 2000). Hermeneutic approaches tend to explore the creation of meaning as an inherently constructivist process, as opposed to an objectivist one. In this study, we used Grounded Theory (Strauss and Corbin, 1998) to make the transition from single-text analysis to the multiple-text analysis. This process simultaneously revealed dominant patterns that constitute super-categories as well as patterns unique to a specific narrative. We sought to examine hidden strata within the text by adopting a hermeneutics of suspicion (Josselson, 2004). Two independent expert clinicians and experienced qualitative researchers worked systematically to code and analyze each testimony. Furthermore, they noted specific instances of the way the testifying women chose to explain their acts or their stance regarding military situations that included excessive violence. Each one of the testimonies were compared with all other testimonies; the “subcategories” identified through this process represent the themes common to all testimonies. Once such subcategories were developed, they were compared with each other to develop upper level “categories.” Continuing this process of comparison created a data hierarchy, wherein “clusters” of categories were the highest level developed. These clusters gathered together the categories that represented the most salient themes. In grounded theory, data collection continues until new data ceases to add new meanings to the hierarchy and “saturation” is reached (Fassinger, 2005). The hierarchy was saturated at the 15th testimony, meaning that no new categories were created while we examined the rest of the testimonies, which in turn indicated that the analysis was comprehensive. Furthermore, all along the analysis we used memos to track and document our understandings of the data (Rennie, 2000); this procedure helped us make sure that data analysis reflected the depth of the phenomenon, as well as its complexity. A process of consensus was used in the creation of categories between the researchers who met twice a month to discuss the analysis for a period of approximately 1 year. This consensus-seeking procedure encouraged the researchers to raise issues for the other to consider. In keeping with the epistemological stance, the researchers sought to recognize each other’s areas of expertise (one has clinical experience working with ex-combatants and the other supervises and trains psychoanalysts) to enhance their combined attunement to the data. Through these discussions, differences in interpretation were resolved.

## 3 Findings

The majority of women (58) discussed their experiences using gendered terms and in relationship to their positionality as women in the army. Further analysis revealed three gendered narrative categories that explain women veterans’ reflections on their actions in the face of violent acts. The categories are conceptualized in terms of the tension between being victims or aggressors. The following are the three categories of women veteran’s responses to excessive violence in combat: 1. Identification with the (male) aggressor, 2. Internalized oppression and learned helplessness, and 3. Feminist resistance.

Most of the women veterans internalized their gender’s oppression, and described their acts as reflecting identification with the hierarchical military system and culture (the first category); others expressed recognition of and outward conformity with the military system without truly identifying with it (the second category). Fewer testimonies presented an attempt to resist while taking an explicitly feminist perspective. In the following section, we describe and analyze each category.

Given the complexity of the issue at hand and the need to fully articulate and interpret the nuances of the testimonies as they are presented in the original and full context, the quotes in the following subsections were excerpted from only four testimonies. Although limiting the amount of evidence presented while expanding on the interpretative analysis creates the impression of an imbalance between “showing” and “interpreting” (Pratt, 2009), the authors view this imbalance as imposed by the publication-related limitations combined with the desire to demonstrate and explain complex phenomena in detail. To further compensate for the small number of excerpted testimonies quoted herein, the percentage of statements that referred to each category, as well as the number of interviewees that these statements represent, are included in the findings, although such a level of detail is not considered mandatory in a qualitative study.

### 3.1 Identification with the aggressor

The first category – (1), which was expressed in 34% (*n* = 20) of the women veteran’s testimonies, described their identifying with male violence and also joining the male combatants in abusing the Palestinians. Such responses gave them a sense of supremacy over the victims. Moreover the female veterans in this category explained that they identified with the aggressor, adopted masculine codes and language, and engaged in violent actions, to equate their status with that of their male peers and thus they also protected themselves from military gender biases. This need likely stems from the tenuous position of women combatants in the IDF, a role which has only recently been sanctioned and is under constant political and discursive criticism and attack.

Ferenczi (1933) and Freud (1936) were the first to write about the phenomenon of trauma-related aggression, called “identification with the aggressor.” Identifying with the aggressor is a wide-ranging, multifaceted process which goes beyond behavioral mimicking of the attacker’s aggression, Lahav et al. (2022). Ferenczi’s theory reveals four main aspects: losing one’s agency and replacing it with that of the aggressor or perpetrator; becoming hypersensitive to the perpetrator; adopting the perpetrator’s experience concerning the abuse, and identifying with the perpetrator’s aggression (Lahav et al., 2019). The testimonies in this category demonstrate all four aspects of identification with the aggressor. The following vignette represents a typical testimony that contains these elements.

The veteran in this interview was one of a small minority of women in an entire battalion. She opened her testimony by mentioning that when she first arrived at the camp, male soldiers yelled out at her: “Fresh meat, fresh meat!” The woman was then subjected to continuous offensive and aggressive behaviors from most of the male soldiers. In her testimony, the veteran describes the military atmosphere as one that does not make independent thinking possible. She not only surrendered to this hostile environment and denied what was happening around her, but further adopted the ethical stance concerning use of violence to the point where she used excessive violence herself:

To see those children in Hebron walking by, and to take pride in the fact that they are afraid… I mean, these are kids, but it had already become a norm. And you can’t figure out whose side you’re on. I’m a Jewish-Israeli soldier, and I’m supposed to be against the Arabs who are my enemies, but I’m here at the outpost, and I think that the Jews are wrong. So, wait a minute, no, I have to change my mind and go on hating Arabs and justifying the Jews.

So why make the switch?

Out of loyalty to your own kind … Yes. And you don’t really think, you just say whatever comes into your mind right then: I hate this guy so I’ll swear at him, and then I hate that one, so I’ll swear at him, and now I hate him, so I’ll spit at him.

You would spit at Arabs?

Well, they’re Arabs, I mean, I don’t know. True, the one specific Arab I spat at didn’t actually do anything to me. Seems to me he’d done nothing at all to anyone. But again, it was a cool thing to do, and the only thing I could do. I mean, I can’t go around boasting of having arrested anyone, or be proud of having caught a terrorist, or killing a terrorist; I can’t go on some mission and find some weapons under a floor tile in a home. But I can spit at them and humiliate and ridicule them (Rank: Sergeant, Unit: Nahal, Location: Hebron)

Freud (1936) described how the victim internalizes the aggressor’s behavior until s/he becomes an aggressor, unleashing her/his aggression on others, who, in turn, become the aggressor’s victims. We can see, from the veterans’ account, how she transformed herself from a victim, who was treated with disdain and whose body was disrespected (male soldiers yelled at her: “Fresh meat, fresh meat!”), into a dismissive aggressor. By spitting on the Palestinian, who, by her own account, did nothing to offend her or other soldiers, she expressed disrespect for the integrity of his body. In a way, she used the body of the Palestinian to create supremacy, just as her male colleagues debased her body, in order to create their sense of supremacy. She justifies her actions by saying that to gain respect she must demonstrate superiority over and subjugation of someone. As the limits of her role do not allow for a typically masculine expression of violence (e.g., “I cannot go around boasting of…”), she concludes that she must find her own way to express it. Thus, this category reveals the impact of an aggressive professional culture, which imbues not only the group’s internal interpersonal interactions with expressions of discrimination and oppression, but it is also projected outward, onto disadvantaged groups. Thus, the inward and outward weaving served to both spread and preserve the culture of violence.

### 3.2 Internalized oppression

The second category – (2), internalized oppression, was found in 40% (*n* = 23) of the testimonies. This category represents women veterans who did not approve of the acts of excessive violence they witnessed, yet they suppressed their moral voice. They explained this choice as related to the realization that they had no impact when facing the dominant and aggressive male soldiers. Consequently, they took on a position of learned helplessness and powerlessness. Thus, this category demonstrates that the women felt forced to conform to masculine language and masculine behavioral codes, without identifying with them. The women used these codes (violent acts) openly, while any attempts to undermine or oppose violent conduct were done in a covert and implicit manner.

The psychological phenomenon of internalized oppression occurs when a person internalizes prejudice and bias concerning the identity of the group(s) to which one belongs (David, 2013). Researchers have linked internalized oppression to many kinds of harm (Cudd, 2006), as people who internalize prejudice and bias tend to perpetuate them in their interactions with others. This idea is further supported by the system justification theory (Jost and Banaji, 1994) which claims that people are motivated to defend, bolster, and justify aspects of existing social, economic, and political systems, even at the expense of individual and collective self-interest. This tendency manifests in phenomena like “the participation by disadvantaged individuals and groups in negative stereotypes of themselves” (Jost and Banaji, 1994, p. 1) and outgroup favoritism, which help maintain existing arrangements despite potential costs. Together, these perspectives suggest that internalized oppression is not only a symptom of an oppressive social climate, but also works as a mechanism that secures its continued existence (David, 2013). The following testimony was selected to demonstrate this category:

There was another border patrolwoman with me. Again, we’re talking about women, so I think the women combatants are more violent than the guys. And she called out to kids: “Come here!” So they climbed the hill. She opened their bags and found this kind of fly-swatter inside. They were scared. You could see that those kids wouldn’t dare refuse. One of the kids tried to smile as he handed it to her. He tried, I don’t know, to be like a kid and believe she was human. Boom! She hit him with that flyswatter. The kid began to cry. She said, “What, you’re crying? Off with you, run up the hill again.” She hassled him about five times. Finally, she turned to me: “Well?” and I answered, “Leave him alone; let him get away from here.”

I quickly understood that it would do no good to try and talk them [the officers] out of it. What is it that I finally figured out? I was scared, and I didn’t believe that if I said to them, “Listen, this is out of line,” it would make a difference to anyone. They weren’t interested and no one would have changed a thing.

I always tried their [male soldiers] language: “Bah, let them go; leave ‘em alone, you shouldn’t even bother with them.” I said all sorts of things, as if I wasn’t at all concerned about the [Palestinians’] condition, but implying that it’s below us to even deal with them. And there were other guys, to whom I said “Listen, why are you beating up this kid? Tomorrow a Hamas member will adopt him and tell him to come and take revenge on that soldier. And why wouldn’t he? But if you treated this kid well? I’m not telling you to let him in if he’s not supposed to go in – just send him home.” (Rank: First Sergeant, Unit: Border Patrol, Location: Seam Zone)

Here the veteran did not dare to openly oppose the abusive behavior of either her female or male superiors (officers), in similar contexts. However, her testimony makes it clear that she did not identify with their behavior. Rather, she tried to “tiptoe between the raindrops”: on the one hand, she avoided declaring her moral stance, while on the other hand, she attempted to stop the abusive behavior toward the children. She camouflaged her original intentions (defending the Palestinian victims), by concealing them behind an alleged attempt to protect the soldiers from the consequences of their own extreme actions. She masked her conscious attempt to stop the abuse by adopting the military behavioral and linguistic codes that her colleagues were using.

### 3.3 Feminine resistance

The third category – (3), feminist resistance, which was found in 26% (*n* = 15) of the testimonies, reflected the female veterans’ critical gendered voice. They used this voice to assert their own moral values in the context of a combat situation. This category represents the female veterans who did not hesitate to fight for their values, using gendered terms. The following two testimonies shed some light on these instances.

The first example:

One day I went out to the gate to hitch a ride. I stood there with the other guys. Across the road there were Palestinian detainees seated. The guys on guard duty at the brigade HQ base were on routine security shifts. They would stand guard at the camp gate. So, as I’m standing there, I see these two guys pacing around a detainee – blindfolded, his hands shackled behind his back. Suddenly I see that one of the guys simply approaches him and, without any warning, knee-kicks him in the head. My gut-reaction was instant: I leapt at this guy, caught him and said: “You’re coming with me now.” He didn’t understand what on earth a woman soldier is doing ordering him around. He shoved me off – this was a big guy – he pushed me away and ran into the barracks. I was all wound up at that point. I left the gate, hurried upstairs straight to the deputy-commander’s office and told him what happened, and he said, “Go with our deputy operations-officer and find that fellow.” I went with the ops-officer, the guy must have hidden somewhere on the base; we couldn’t find him. When we returned to the deputy-commander and told him we hadn’t found him, he said: “Let it go, it’s pointless. Anyway, there are and there will be many such cases,” or something in that vein. I don’t recall his exact words, but that was the message: “Let it go, no need to pursue this.” It was one of the instances I had a very hard time with (Unit: Hebron Regional Brigade/Education Corps Officer, Place: Hebron)

Despite her rebellion and determination to convince the higher ranks to restore justice, the fact that the male soldier was physically larger than her had allowed him to escape (not before he expressed his surprise that a women soldier had confronted him). Eventually, her superiors’ refusal to act caused her to lose spirit.

In another testimony, we see once again an attempt by a women soldier to change the military’s norms and the predominant approach toward Palestinians, from inside the system. However, in this case, the steep price that she had to pay for standing up for her values, was revealed:

There were Palestinians walking with their carts and wagons and donkeys on the roadside, some on foot. The Border Patrolmen, who were in the truck, took the left-over food that was in the crates in the truck and began to throw it at the Palestinians. It was my most shocking experience in the Occupied Territories. I got hysterical, and I yelled at them: “What are you doing?! What’s going on with you?” They only laughed and forced me back into my seat: “Sit down.”

I knocked on the cabin door, to get the officer’s attention and demanded that the truck stop immediately and not go on driving. The officer told me to be quiet, “Don’t interfere.” Yelling, I described what was happening: “They’re throwing vegetables at Arabs! Stop!” They didn’t stop the truck, they didn’t mind it, they did nothing.

I got to the base extremely upset. I had a talk with the unit commander. He didn’t seem too perturbed: “Forget it.” I talked to other people there and no one … Everyone tried to hush it up somehow.

I remember that after this whole storm, after I tried to talk to all the commanders and no one wanted my input, then they no longer paid any attention to me at all. They really avoided me … It was really difficult. Especially realizing how small I was, although I felt that I ought to be strong enough [to do something about it]. It was very hard.

All those feelings there; it wasn’t new; every Saturday night I would cry my heart out at home. Their male chauvinism, the incessant humiliation, especially this constant power-play; feeling that I was the weakest, weirdest creature in the world, complaining all the time (Rank: Sergeant, Unit: Border Patrol, Location: Gaza Strip)

Here the veteran presents an explicit connection she makes to the gendered dynamics in the army, which impacted her actions and feelings (“Chauvinism,” “humiliation,” “power-play”).

Both of these testimonies reveal women in revolt – unafraid of the price they might have to pay for protesting. Both women explicitly protected the trampled moral values and were willing to pay the price of isolation and exclusion from the group. The use of the term “hysterical,” in the second testimony, is interesting, mainly because this so-called diagnosis is often used as an oppressive mechanism against women. This usage demonstrates the work of re-appropriation: while understanding that she would be categorized as “hysterical,” because she reacted “abnormally” to what others perceived as normative, she understood these norms to be pathological (she yelled on the soldiers: “What’s going on with you?”). Therefore, the way to protect her humanity was to protest the violent behaviors. Even though she initiated and led the fight, she concluded her testimony with feelings of loneliness and alienation, as if something was wrong with her.

### 3.4 Testifying within the military

The three categories offer an explanation as to how women soldiers in combat areas experience and try to navigate the complicated dynamics and opposing pressures of being both oppressed within the military setting while being in powerful, aggressive roles. As seen elsewhere (Stander and Thomsen, 2016), women in this study described different types of oppression within the context of military service. In particular they mentioned experiencing chauvinistic norms and behaviors, professional marginalization through sexualized humiliation, sexual harassment from fellow soldiers, and sexual harassment from Palestinian detainees.

Although all of the testimonies described an environment of oppression within the military, we note a difference between the categories in terms of the frequency of expressions used to convey their feeling of being oppressed by and within the system. Among the women whose testimonies fall under the category of identification with the aggressor, only 20% of them described specific situations in which they experienced oppression during their service. In contrast, 70% of the women whose testimonies were categorized as “internalized oppression,” and 67% of the women whose testimonies were categorized as examples of female resistance, described specific instances of oppression. This choice to describe specific instances of oppression emphasizes the degree to which these female soldiers experienced the military as a toxic environment, in which women must cope with violence and oppression.

Women whose testimonies were categorized as “identifying with the aggressor” were less likely to describe the military framework as a toxic and oppressive environment. One explanation is that this “identification” mechanism helped them suppress – in their subjective experiencetheir awareness of the oppression directed toward them. Another possibility is that they found that this mechanism effectively lessened the extent of oppressive behaviors directed toward them, even if at the cost of ignoring their moral standards and hurting others.

## 4 Discussion

This study investigated the ways women veterans of combat or combat-support units conceptualize their stance regarding violent acts which they either committed or witnessed in war zones. Using the retrospective testimonies of 58 Israeli women veterans it was revealed that most women explained their violent acts as inherent to the military system and culture, which in our analysis was categorized as examples of either internalized gender oppression or as identification with the aggressor. A smaller number of women described their attempts to protest, as they took a moral stance rooted in a feminine perspective.

The women in this study, who testified about their military experiences in war zones, were not asked directly about gender oppression; they brought up the topic spontaneously. This fact highlights the centrality of their gendered oppressive experience in the military. It coincides with and supports the results of a previous study, which highlighted the different perceived stressors reported by men and women following a war-zone deployment period (Street et al., 2013). Women’s stressors included increased risk of sexual harassment, general harassment, poorer unit support, as well as an increased risk of depression (Koeszegi et al., 2014; Harel-Shalev et al., 2017; Steidl and Brookshire, 2019). On the other hand, this study validates the observations made by Abraham et al. (2017) and Ramon et al. (2020), which emphasized the deeply ingrained masculine ideologies within military institutions that prize strength, dominance, and emotional control. The testimonies from the women veterans in our study reveal how these ideologies shape not only the culture but also the individual identities and responses to violent situations, highlighting the internalization of gender oppression as a mechanism of coping within a masculinized military environment. Moreover, our findings resonate with the work of Neilson et al. (2020) and Zerach (2023), who identified the mental health challenges faced by women due to the pressure to conform to traditional masculine norms through maladaptive coping strategies. One of the study’s main findings is that for women serving in the military in positions that entail policing and doing combat, being subjected to oppression and humiliation by their fellow male soldiers adds psychic and social difficulties to an already complex reality. The study adds to former research in the field by linking women’s military experiences, specifically, their participation in and reaction to violent events they have witnessed, to their experience of being subjected to gendered oppression.

The focus of our analysis was on the connection between women as an oppressed minority in the military and their reaction to morally fraught situations. The findings of Held et al. (2019) showed that contextual factors, such as situational chaos, power, rank, and perceived need to prove oneself, are likely to affect soldiers’ decision-making process in morally injurious situations. Our study further confirmed this finding by demonstrating that gender, which in the military is related to power, self-perception, and oppression, also affected the female soldiers’ decision-making process in their reactions to excessive violence. However, in contrast to Held et al.’s (2019) study, gender cannot be considered a contextual factor. Rather, as shown, it has a critical effect on soldiers’ military experience. In this manner, the current study contributes to the literature on soldiers’ emotional reactions to violent situations, by showing how gender dynamics play a central role in shaping women soldiers’ responses.

Three categories emerged from our analysis of women veterans’ testimonies, in which they explained their participation in or responses to acts of excessive violence. A common feature of all three categories was their being forced to choose whether to act as aggressors or become victims of their peers’ aggression.

Their responses reflected a range of psychological mechanisms: from identifying with the aggressor, which led the women to become abusers themselves, to internalizing the gender oppressing views expressed by their male counterparts, which led them to adopt a learned helplessness that prevented them from resisting, despite feeling morally outraged by the injustices they witnessed. Finally, some women expressed overt resistance, by explicitly protesting against the injustices they witnessed. This resulted in the women paying a heavy price of isolation and exclusion for their resistance.

Our finding regarding the female veterans who either joined in violent acts or condoned such acts committed by others (categories 1 and 2) supports the findings of a previous study concerning male veterans, which showed that a pattern of aggressive and abusive behaviors may become normative over time (Ben-Ari, 1998). Similarly, the male veterans perceived violent acts as a way of fitting in, creating a sense comradery, and building trust with others (Held et al., 2019). Considered from a different perspective, our findings also coincide with those of Iverson et al. (2012), which demonstrated that during military training, soldiers develop a strong, tough, and physically powerful identity. In addition, traditional masculine norms, such as self-reliance, toughness, and restrictive emotionality, are known to inhibit the cognitive-emotional processing of traumatic events (Lorber and Garcia, 2010). Therefore, women who were subjected to gender oppression during their military service would be likely to reject this position as victim and would opt instead to reclaim their control, by adopting masculine-identified power behaviors, specifically those expressed through violent acts. Alarmingly, this finding demonstrates that instead of fostering a sense of unity and shared mission, military training may inadvertently intensify feelings of marginalization and powerlessness among female soldiers.

This line of thought also reflects and expands on Sasson-Levy’s (2003) argument, namely, that Israeli female soldiers serving in traditionally masculine roles effectively shape their gender identity to coincide with the hegemonic perception of the combat soldier as masculine, which they achieve through mimicry of combat soldiers’ bodily and discursive practices, distancing from traditional femininity and trivialization of sexual harassment. The practices identified by Sasson-Levy can be said to coincide with Category 1 in our study. On a theoretical level, our study contributes to the field by conceptualizing two additional strategies that female soldiers use (Categories 2 and 3), which have important psychological and clinical implications.

Thus, for example, the strategy of internalized oppression can be contextualized by noting that military training typically instills in soldiers the understanding that they are currently property of the military and, as such, are required to sacrifice individuality for a perceived greater good (Smith and True, 2014). However, as the current study reveals, instilling this perception within the military context, where masculinity is still the dominant gender and gender oppression is still the norm, is likely to be counterproductive for the effective integration of female soldiers.

The third strategy conceptualized in this study, that of resistance to excessive violence, derives from a conscious feminist position, and in this sense, it echoes the findings of the study by Karazi-Presler et al. (2018) on Israeli women officers. The study reported that female officers conveyed a dialectical and emotionally complex experience of power, which they experienced as a source not only of pleasure and empowerment but also of shame. The researchers argued that this reflects the inherent gender boundaries of military power and women’s place in the military power hierarchy. In our study, women veterans who testified about their resistance described feeling trapped – between being faithful to their moral values, on the one hand, and fearing the social price of challenging the system’s gender norms, on the other.

However, this pattern of resistance can be considered in terms of the findings reported by Evans et al. (2018), which showed that although veterans who connect with and engage in practicing their values actively may experience greater distress related to acts of injustice they committed, they are also likely to experience greater overall life satisfaction, because of that same strong and deep commitment to their values. In other words, in line with Evans et al. (2018), the female soldiers who paid a price for their resistance during their military service because they favored their individual values (which in fact coincide with the military’s official code of ethics) instead of succumbing to commonly practiced norms could, in the long run, experience a positive sense of well-being, despite the mental and emotional anguish it cost them to uphold their values during their military service. In this context, it should be noted that, although inadvertently, these women’s struggles to adhere to the moral code in effect contribute to the military’s superior performance.

This study joins other numerous examples found in the literature on the price women pay for being non-conformists, whistle-blowers, resisters, or acting assertively (e.g., Harel-Shalev and Daphna-Tekoah, 2016). It also joins the body of research that focused on the resistance to hegemonic masculinity within the military that highlights a potential shift toward a more inclusive understanding of masculine identity (Robinson Kurpius and Lucart, 2000; Perez and Sasson-Levy, 2015; Smith et al., 2019; Pendlebury, 2020; van Douwen et al., 2022). As such, it presents a complex picture of women’s struggle for gender integration in the military and the costs associated with it. Creating spaces within the military for men to explore and express a broader range of masculinities can contribute to a more inclusive and supportive environment for all. This includes challenging the notion that certain qualities or roles are inherently masculine or feminine and promoting the idea that strength comes in many forms.

This study has an important and unique theoretical and clinical contribution, as the women in each of the three categories incur a social and/or mental cost for the strategy they use for adjusting to the violence that characterizes the military milieu. Those in Category 3, who go against the hierarchy and the double violence (violence both from the soldiers toward them and from the army and toward the Palestinian population) are socially and professionally marginalized. Those in Categories 1 and 2, who seek to integrate and assimilate into the military milieu lose touch with their morality, as evidenced by their decision to testify and thus “break the silence.” Furthermore, as mentioned, those in Category 2, who internalized the oppression and remained silent, expressed mental distress more than those who used the other two strategies, probably because they gained neither the empowerment of protest nor the illusory power of militarism.

These findings coincide with the findings of previous studies, which showed that military culture increases and emphasizes emotional detachment and leaves military personnel to deal with difficult emotions through suppression (Nash et al., 2009; Vogt et al., 2011). The lack of recognition from significant others is coupled with colleagues’ disapproval and rejection from the larger social milieu, while anxiety and emotional discomfort are consistently labeled “irrelevant.” It was also found that former-combatants who held traditionally masculine attitudes were prone to greater vulnerability in the aftermath of traumatic events and coped poorly with such events (Ramon et al., 2020). In the current study, we suggest that women who have adopted similar attitudes toward violence may also suffer from similar symptoms. Thus, this conclusion urges us to critically reassess the current approach toward military training, especially with respect to fostering a highly masculine-image of power and strength. It also highlights the need to implement more supportive measures to address and prevent victimization within the military environment.

One might inquire whether, by describing the women as witnessing and reacting to male violence rather than as active agents who engage in violence of their own accord, we might be casting them in a passive role, much as we claimed that previous studies have done. Why are men considered the aggressors whereas women are described as joining in only because they identified with the aggressors? Might not women be aggressive too? Presumably that too is possible; however, the current research focused on the way women veterans subjectively conceptualized and interpreted their behaviors while serving in a combat zone, reassessing the stance they took regarding morally fraught situations.

The choice of a stance regarding violent acts which they either committed or witnessed in war zones can be highly individualized and contextual, depending on the person’s unique experiences, personality, and resources. A few studies have reported a connection between belonging to a combat unit and increased mental resilience, demonstrating that meeting challenges as part of a team can mediate the coping experience, and thus successful coping can give combatants a sense of meaning, which in turn creates an association between combat service and heroism (Lepore and Revenson, 2006; Green et al., 2010). However, as shown, women serving in a predominantly male combat unit are often not treated as part of the team but rather relegated to a position of inferiority. At the same time, they may be feeling empowered, by virtue of being female fighters. An important contextual variable that stood out in our study was gendered oppression characterized by chauvinist attitudes, which led women combatants to choose one of the two defensive stances, identifying with the aggressor or internalizing the oppression, rather than the third stance characterized by resistance.

Methodologically, the article offers new paths to studying marginalized and silenced voices of women in the military, by using interviews that were carried out by women who were in combat or combat-support roles themselves. It is possible that this fact enabled the women interviewees to honestly and openly share their experiences regarding the use of excessive violence, an issue that had not been raised in previous studies.

### 4.1 Limitations and future research

This study has some limitations that should be addressed. We acknowledge the need to consider the timeline of testimonies and how the evolving military context may influence the experiences of women veterans. This reflection opens avenues for future research to explore changes in military culture and their impact on gender dynamics over time. This study did not examine the association between the categories and other variables, such as ongoing exposure to the risk of death or injury, which are known to affect narrative style and form, nor did it check previous levels of trauma, another factor that could affect and explain soldiers’ aggressive behaviors in the aftermath of combat deployment (Richardson et al., 2010).

The complexity of women’s experiences, especially for those who navigate tensions between multiple strategies or grapple with conflicting roles, warrants further investigation. This aspect of individual variability was not fully explored, underscoring the need for a more detailed exploration of these nuances. Moreover, this study alludes only briefly to the possibility that participation in the “Breaking the Silence” archive serves as a distinct strategy for managing the impact of exposure to excessive violence, noting that such participation can provide a confessional outlet which results in personal moral relief. However, this possibility raises important questions about the potential role of such participation in shaping personal narratives and coping mechanisms. Therefore, future research should aim to delve into these individual differences more profoundly and consider the psychological implications of engaging in initiatives that document experiences of violence, enhancing our understanding of the unique challenges faced by women in military settings. It also would be interesting to see if and how the collection of veterans’ testimonies in an independent research context might impact the types of categories discerned. Future research should also aim to identify strategies for cultivating an inclusive military environment that challenges and redefines traditional gender norms affecting both women and men. This includes studying ways to reconceptualize virtues like strength, courage, and leadership beyond gender stereotypes, promoting diversity of identity and capability among all service members. Additionally, it is critical to investigate how to support and protect individuals challenging these norms, ensuring they are not marginalized. Such research would offer insights into creating a military culture where diversity and inclusion benefit every member, regardless of gender.

Finally, the study’s findings reveal that testimonies about acts of excessive violence in the military can serve as a platform that allows us to observe individuals’ internal negotiations between conflicting inner forces, experiences, and worldviews. These categories may serve as a key tool for deciphering gender dynamics; identifying and understanding gender-embedded coping practices could offer new directions for future research.

## Data availability statement

Publicly available datasets were analyzed in this study. This data can be found at: https://www.breakingthesilence.org.il/wp-content/uploads/2011/02/Women_Soldiers_Testimonies_2009_Eng.pdf.

## Ethics statement

Ethical approval was not required for the study involving humans in accordance with the local legislation and institutional requirements. Written informed consent to participate in this study was not required from the participants or the participants’ legal guardians/next of kin in accordance with the national legislation and the institutional requirements. Written informed consent was obtained from the individual(s) for the publication of any potentially identifiable images or data included in this article.

## Author contributions

NH: Conceptualization, Investigation, Methodology, Project administration, Writing – original draft, Writing – review & editing. DA: Supervision, Writing – review & editing, Conceptualization, Investigation.
